# Calcium-dependent protein kinases as central hubs in plant abiotic stress signaling: mechanisms and prospects for crop improvement

**DOI:** 10.3389/fpls.2025.1711405

**Published:** 2026-01-30

**Authors:** Samuel Azupio, Yu Wang, Joshua Obeng, Abdul Kadir Issah, Jing Chu, Hongmei Zhang, Qing Xie, Xingyu Jiang

**Affiliations:** 1 College of Coastal Agricultural Sciences, Guangdong Ocean University, Zhanjiang, China; 2 National Center of Technology Innovation for Saline-Alkali Tolerant Rice, College of Coastal Agricultural Sciences, Guangdong Ocean University, Zhanjiang, China; 3 Council for Scientific and Industrial Research – Oil Palm Research Institute, Kade, Ghana; 4 Department of Plant Science, College of Agriculture, Tennessee State University, Nashville, TN, United States

**Keywords:** abiotic stress, calcium-dependent protein kinases, climate-resilient crops, phosphorylation, reactive oxygen species, plant stress response, cross-talk, transcription factors

## Abstract

Calcium-dependent protein kinases (CDPKs) are key Ca²^+^ sensors that decode stress-induced calcium signatures through substrate phosphorylation, ROS regulation, kinase cross-talk, and precise spatiotemporal activity, playing a central role in orchestrating plant responses to abiotic stress. This review systematically examines the central role of CDPKs in mediating plant tolerance to drought, salinity, and extreme temperatures. It highlights how CDPKs regulate key processes such as stomatal closure, ion homeostasis, osmotic balance, antioxidant defense, and gene expression through the phosphorylation of transcription factors, ion channels, and metabolic enzymes. Moreover, it discusses the functional redundancy and specificity within large CDPKs gene families, which enable precise responses to specific stresses. Furthermore, the review explores the critical cross-talk between CDPKs signaling networks and phytohormone pathways, particularly abscisic acid (ABA), and their integration with reactive oxygen species (ROS) and MAPK signaling cascades. Recent advances demonstrating the potential of manipulating specific CDPKs isoforms to enhance multi-stress resilience in transgenic plants are also summarized. By synthesizing current knowledge, this review provides insights into the molecular mechanisms of CDPKs-mediated stress adaptation. It identifies future research directions for developing climate-resilient crops through targeted genetic engineering of CDPKs signaling pathways.

## 1 Introduction

Plants are subjected to various environmental stresses, such as drought, heat, salinity, and low temperatures, which prevent their growth and reduce productivity (Zhang et al., 2023). They engage in signaling pathways and regulatory processes that sense the stress signals and react by releasing responsive genes with the help of adaptive biochemical and physiological responses (Kim et al., 2021).

At the core of this adaptation is calcium, a second messenger that encodes environmental stress signals through its fluctuations. These Ca²^+^ dynamics are sensed and interpreted by Calcium-dependent protein kinases (CDPKs), which act as pivotal molecular transducers, translating calcium oscillations into phosphorylation-driven signaling events that regulate gene expression, ion transport, and metabolic reprogramming (Dekomah et al., 2022). The calcium sensors, such as CDPKs and calmodulins, calcineurin B-like proteins (CBLs), perceive the stress-induced changes in cytoplasmic calcium and subsequently activate more targets in these pathways (Ren et al., 2023) and participate in hormonal, stress, and metabolic cross-talk, leading to rapid stress adaptation (Yip Delormel and Boudsocq, 2019). CDPKs are unique serine/threonine kinases harboring an intrinsic calmodulin-like regulatory domain that enables them to directly sense and decode intracellular calcium signals without requiring separate calmodulin proteins (Boudsocq and Sheen, 2013; Valmonte et al., 2014; Yip Delormel and Boudsocq, 2019). CDPKs also contain at least four domains, including a variable N-terminus domain (VNTD), a protein kinase catalytic domain, and an autoinhibitory junction domain (AI-JD) and C-terminal calmodulin-like domain (CaM-LD) harboring EF hand motifs for calcium perception; the structures of these latter domains have been resolved from several plant species, including *Arabidopsis thaliana*, *Glycine max* (Cheng et al., 2002; Liese and Romeis, 2013; Xu et al., 2015). This VNTD varies greatly between CDPKs and serves as a site for functional specialization and subcellular localization with the N-terminal myristoylation and palmitoylation, leading to the specific interactions with certain membranes or substrates (Boudsocq and Sheen, 2013; Shi et al., 2018).

For example, *AtCPK6* regulates the Na^+^/H^+^ antiporter SOS1 to enhance salt tolerance in *Arabidopsis thaliana*, and *AtCPK23* mediates drought-induced stomatal closure (Quan et al., 2007; Brandt et al., 2012). Conversely, *AtCPK23* modulates stomatal closure by phosphorylating slow anion channels (SLAC1), a key mechanism for limiting water loss during osmotic stress (Mori et al., 2006). In rice (*Oryza sativa*), *OsCPK21* preferentially interacts with bZIP transcription factors to induce drought-responsive genes, while *OsCPK4* regulates MAPK pathway components under combined drought-salt stress (Tanpure et al., 2021; Geng et al., 2024). The binding of Ca^2+^ to the CaM-LD causes a release of autoinhibition (AI). It therefore evicts AI-junction domain residue from its inhibitory site on the enzyme-capping junction domain, activating kinase activity (Liese and Romeis, 2013; Yip Delormel and Boudsocq, 2019; Bredow and Monaghan, 2022). Therefore, the variations in the EF-hand motifs and calcium-binding affinities give rise to different activation kinetics among CDPKs isoforms that further increase the diversity of their signaling functions (Yip Delormel and Boudsocq, 2019). CDPKs target substrates such as ion channels and transcription factors, translating calcium signals into stress, development, and hormonal responses (Mittal et al., 2017; Marques et al., 2022). Gene duplication and divergence have led to the expansion of CDPKs families for plant-specific functions in species including *Arabidopsis*, rice, and tomato (Xu et al., 2015). **Table 1** provides a comparative overview of CDPKs gene distributions across major crops and model plants, illustrating both conserved and lineage-specific expansions that underpin functional specialization in stress signaling, development, and metabolic regulation. This analysis shows that expanding these families due to variations in domains is important for plant adaptation mechanisms.

**Table 1:** CDPKs Distributions among specific plants.

Common name	Botanical name	No. of CDPKs	Genome size (Mb)	Key functional differentiation/feature	Notable isoforms/genes	References
Apple	*Malus domestica*	28	881.3	Subcellular localization diversity is involved in stress and development.	*MdCPK1, MdCPK6*	(Mohanta et al., 2015; Dong et al., 2020)
Alfalfa	*Medicago sativa subsp. sativa*	38	800	CDPKs involvement in nitrogen fixation, drought, and salt stress signaling.	*MsCPK1, MsCPK10*	(Han et al., 2024)
Banana	*Musa acuminata*	44	523	Diverse isoforms showing tissue-specific expression under abiotic stress.	*MaCPK7, MaCDPK3*	(Wang et al., 2016)
Barley	*Hordeum vulgare*	28	667	CDPKs are classified into phylogenetic groups with specialized functional roles.	*HvCPK2, HvCPK4*	(Fedorowicz-Strońska et al., 2017; Yang et al., 2017)
Barrel clover	*Medicago truncatula*	11	360	Limited CDPKs members; focus on symbiosis and stress signaling.	*MtCDPK1, MtCDPK6*	(Ivashuta et al., 2005; Mohanta et al., 2015)
Black cottonwood	*Populus trichocarpa*	28	422.9	Expansion with diverse expression is possibly linked to perennial growth traits.	*PtCPK10, PtCPK25*	(Zuo et al., 2013; Mohanta et al., 2015)
Canola	*Brassica napus*	25	1130	Gene duplication linked to specialized CDPKs functions.	*BnCPK23, BnCPK12*	(Zhang et al., 2014; Lu et al., 2022)
Cassava	*Manihot esculenta*	26	532.5	CDPKs involved in drought and salt tolerance; isoform-specific expressions.	*MeCPK2, MeCPK5*	(Hu et al., 2016; Xiao et al., 2017)
Caster bean	*Ricinus communi*	15	400	Limited CDPKs; functional studies are sparse.	*RcCPK1, RcCPK2*	(Mohanta et al., 2015; Fedosejevs et al., 2016; Ying et al., 2017)
Chinese liquorice	*Glycyrrhiza uralensis*	23	379	The CDPKs gene family is involved in secondary metabolism and stress response.	*GuCPK4, GuCPK8*	(Tong et al., 2019)
Cotton	*Gossypium hirsutum*	98	2250–2430	Significant CDPKs expansion linked to fiber development and diverse stress responses.	*GhCPK6, GhCPK23*	(Gao et al., 2018)
Cucumber	*Cucumis sativus*	19	323.99	Functional divergence in fruit development and Stress signaling.	*CsCPK9, CsCPK3*	(Xu et al., 2015; Zhang et al., 2017)
Garden strawberry	*Fragaria xananassa*	42	692-825	Isoforms participate in fruit development and Stress tolerance.	*FaCDPK4, FaCDPK10*	(Crizel et al., 2020; Mao et al., 2023)
Green bean	*Phaseolus vulgaris*	25	521.1	Functional roles in root development and symbiosis; specialized isoforms in stress response and nodulation.	*PvCPK15, PvCPK3*	(Mohanta et al., 2015; Mallick et al., 2022)
Maize	*Zea mays*	35	2500	Phylogenetic groups correspond to distinct roles in stress and growth regulation.	*ZmCPK11, ZmCPK25*	(Szczegielniak et al., 2012; Ding et al., 2013; Ma et al., 2013)
Melon	*Cucumis melo*	18	375	Specialized CDPKs for fruit development and abiotic stress pathways.	*CmCPK2, CmCPK6*	(Zhang et al., 2017)
Mustard	*Brassica rapa*	49	283.8	Gene duplication results in sub-functionalization in stress response.	*BrCPK13, BrCPK25*	(Mohanta et al., 2015; Wu et al., 2017)
Oat	*Avena sativa*	60	Unspecified	Functional roles in abiotic stress tolerance, especially drought.	*AsCPDK5, AsCDPK12*	(Li et al., 2024)
Orange	*Citrus sinensis*	24	319	CDPKs linked to fruit development and defense signaling.	*CsCPK5, CsCPK9*	(Mohanta et al., 2015; Shu et al., 2020; Zhang et al., 2024)
Papaya	*Carica papaya*	15	135	Roles in fruit development and abiotic stress response; limited but conserved CDPKs functions.	*CpCDPK4, CpCDPK8*	(Mohanta et al., 2015; Zou et al., 2022)
peach	*Prunus persica*	17	206-239.34	CDPKs genes are linked to fruit ripening and stress response pathways.	*PpCDPK2, PpCDPK8*	(Zhao et al., 2022; Vodiasova et al., 2024)
Pecan	*Carya illinoinensis*	31	Unspecified	Limited functional data; presumed roles in abiotic stress tolerance.	*CiCDPK3, CiCDPK9*	(Wang et al., 2025)
Pineapple	*Ananas comosus*	17	526	Functional studies are limited; probable roles in stress response.	*AcCPK3, AcCPK7*	(Zhang et al., 2020; Zhou et al., 2024)
Poplar	*Populus trichocarpa*	30	390-500	The CDPKs family expanded with diverse stress responsiveness and woody perennial-specific functions.	*PtCPK10, PtCPK25*	(Tuskan et al., 2006; Mohanta et al., 2015; Gao et al., 2025)
Potato	*Solanum tuberosum*	23	800	Four major phylogenetic groups; isoforms mediate drought and salt tolerance.	*StCDPK1, StCDPK25*	(Santin et al., 2017; Gromadka et al., 2018)
Rice	*Oryza sativa*	30	372	Well-studied CDPKs subgroups regulate biotic and abiotic responses, including salt tolerance.	*OsCPK12, OsCPK13*	(Ray et al., 2007; Asano et al., 2012; Marques et al., 2022)
Rubber tree	*Hevea brasiliensis*	30	1332	Functional diversification linked to latex production and stress responses.	*HbCPK22, HbCPK8*	(Xiao et al., 2017; Zhu et al., 2018)
Sorghum	*Sorghum bicolor*	28	697.5	CDPKs are involved in drought tolerance and pathogen defense.	*SbCPK9, SbCPK14*	(Mohanta et al., 2015; Mittal et al., 2017)
Soybean	*Glycine max*	50	1115	Diverse stress response functions; some isoforms regulate nodulation.	*GmCPK10, GmCPK25*	(Hettenhausen et al., 2016; Veremeichik et al., 2022)
Thale Cress	*Arabidopsis thaliana*	34	135	Domain variation correlates with substrate specificity, salt, and drought stress signaling.	*AtCPK6; AtCPK23*	(Ronzier et al., 2021; Dekomah et al., 2022; Jafar et al., 2022)
Tobacco	*Nicotiana tabacum*	15	323.75	CDPKs regulate defense responses and abiotic stress.	*NtCPK1, NtCPK2*	(Mee Yoon et al., 1999; Ishida et al., 2008; shuai et al., 2009)
Tomato	*Solanum lycopersicum*	29	900	CDPKs isoforms regulate stomatal movement, reactive oxygen species signaling, and tolerance to biotic stress.	*SlCPK10, SlCPK21*	(Hu et al., 2016; Wang et al., 2016)
Upland Cotton	*Gossypium hirsutum*	98	~2,300	Expanded CDPKs family linked to fiber development and abiotic stress response; functional divergence in stress and hormone pathways.	*GhCPK6, GhCPK23*	(Gao et al., 2018)

This table summarizes the occurrence, genomic distribution, and significant functional features of Calcium-dependent protein kinases (CDPKs) identified in diverse crop and model plants. For each species, the common and botanical names, number of CDPKs genes, and genome size (in megabases, Mb) are provided, along with key notes on functional differentiation, such as stress specificity. The column *Notable Isoforms/Genes* lists representative CDPKs characterized for their distinct regulatory or physiological roles, while *References* cite genome-wide or functional studies that confirmed these findings.

The structural arrangement enables a rapid response to changes in the concentration of Ca^2+^ in the cell, and the binding of calcium activates CDPKs, which undergo auto-phosphorylation and then phosphorylate proteins, transcription factors, and enzymes (Durian et al., 2020; Yang et al., 2021). The kinases are important positive regulators for ABA-mediated drought and salt tolerance because they phosphorylate ABF/AREB transcription factors, promoting the expression of adaptive genes in response to Stress (Soma et al., 2021). They are also required to maintain ion homeostasis, as one of their targets is the toxic ion Na^+^, which is removed from cells by Na^+^/H^+^ antiporters to counter ionic toxicity generated by alkalinity (Zhao et al., 2021). Besides their role in ion homeostasis, CDPKs interact with ROS-related pathways by regulating NADPH oxidases (Ravi et al., 2023; Hao et al., 2024; van Dieren et al., 2024). This regulation influences ROS generation while enhancing the activity of antioxidant enzymes to mitigate oxidative stress (Hu et al., 2020). Furthermore, CDPKs interact with MAPK cascades and hormonal networks, such as the jasmonic acid pathways, to which they are linked during wound signaling, ensuring an integrated and coordinated response to environmental stress (Vega-Muñoz et al., 2020; Kiselev and Dubrovina, 2025).

CDPKs aid in managing essential processes through this phosphorylation, including maintaining membrane stability, regulating osmotic balance, and providing antioxidant defense (Kiselev and Dubrovina, 2025). In addition, the expression and activity of various CDPKs isoforms are preferentially induced due to specific environmental stresses, such as salinity and drought, which may have an inhibitory consequence, as shown in **Table 2**, which summarizes the range of abiotic stress types regulated by CDPKs across different crop species, highlighting both conserved signaling components (e.g., drought- and salt-responsive CDPKs) and species-specific regulatory mechanisms that underlie stress adaptation. Some isoforms have specific functions, while others present redundant or partially overlapping functions or activities (Crizel et al., 2020). Further research is needed to deepen our understanding of CDPKs signaling and develop more effective strategies for enhancing plant tolerance in a rapidly changing environment. Stressing the importance of CDPKs in modulating plant adaptation to complex environments, this article extensively explores the molecular processes associated with CDPKs function and the activation of stress-related genes. As key components of the isonomic network, they are excellent candidates for biotechnological techniques that enhance crop resilience.

**Table 2:** CDPKs involvement in abiotic stress impacts on plants.

Stress type	Impact on plant	CDPKs genes	Reference
Drought	Stomatal closure, reduced photosynthesis and nutrient uptake, and Damage cellular structure.	*OsCPK4*, *OsCPK12* in rice; *ZmCPK4* in maize, *GhCDPK60* in cotton, *AtCPK1, 8, 10, 11* in *Arabidopsis*, *StCDPK3, 5, 13, 23* in potato.	(Das and Pandey, 2010; Vivek et al., 2013; Mittal et al., 2017; Bi et al., 2021; Yan et al., 2022)
Salinity	Osmotic stress disrupts ion homeostasis and toxicity, affecting plant growth and yield.	*AtCPK1*, *3*, *6*, *10*, *11*, *27* in *Arabidopsis*; *SlCDPK2* in tomato, *StCDPK2* in potato, *OsCPK21* in rice	(Asano et al., 2012; dos et al., 2012; Vivek et al., 2013; Grossi et al., 2022),
Extreme Temperatures (Heat/Cold)	High temperatures can cause protein denaturation and membrane damage (organ damage and failure, disrupting the typical structure and function). In contrast, low temperatures can lead to ice crystal formation and cellular disruption (cell death, reduced water availability).	*GhCPK6* in cotton; *TaCPK2* in wheat, *DoCDPK20* in yam.	(Li et al., 2008; Gao et al., 2023; Li et al., 2023),
Oxidative Stress	ROS accumulation damages lipids, proteins, and DNA.	*StCDPK2, 4* in potato.	(Gromadka et al., 2018; Grossi et al., 2022)
Heavy Metal	Disrupt cellular homeostasis, generate ROS, and interfere with enzyme functions.	*OsCPK24* in rice, *AtCPK6* in *Arabidopsis*.	(Jalmi et al., 2018; Keyster et al., 2020)
Flooding/Hypoxia	Affects root respiration, leading to energy depletion and reduced nutrient uptake	*OsCPK21* in rice, *ZmCPK11* in maize, *NtCDPK2* in tobacco	(Witte et al., 2010; Asano et al., 2011; Michael et al., 2023)

This table provides an overview of representative CDPKs genes that mediate plant responses to different abiotic stresses, including drought, salinity, temperature extremes, oxidative stress, heavy metal toxicity, and flooding. For each stress category, the table summarizes the physiological and biochemical impacts on plants, the key CDPKs isoforms or gene families experimentally associated with tolerance mechanisms, and the supporting literature references.

## 2 Role of CDPKs genes in plant drought response

**Figure 1:**
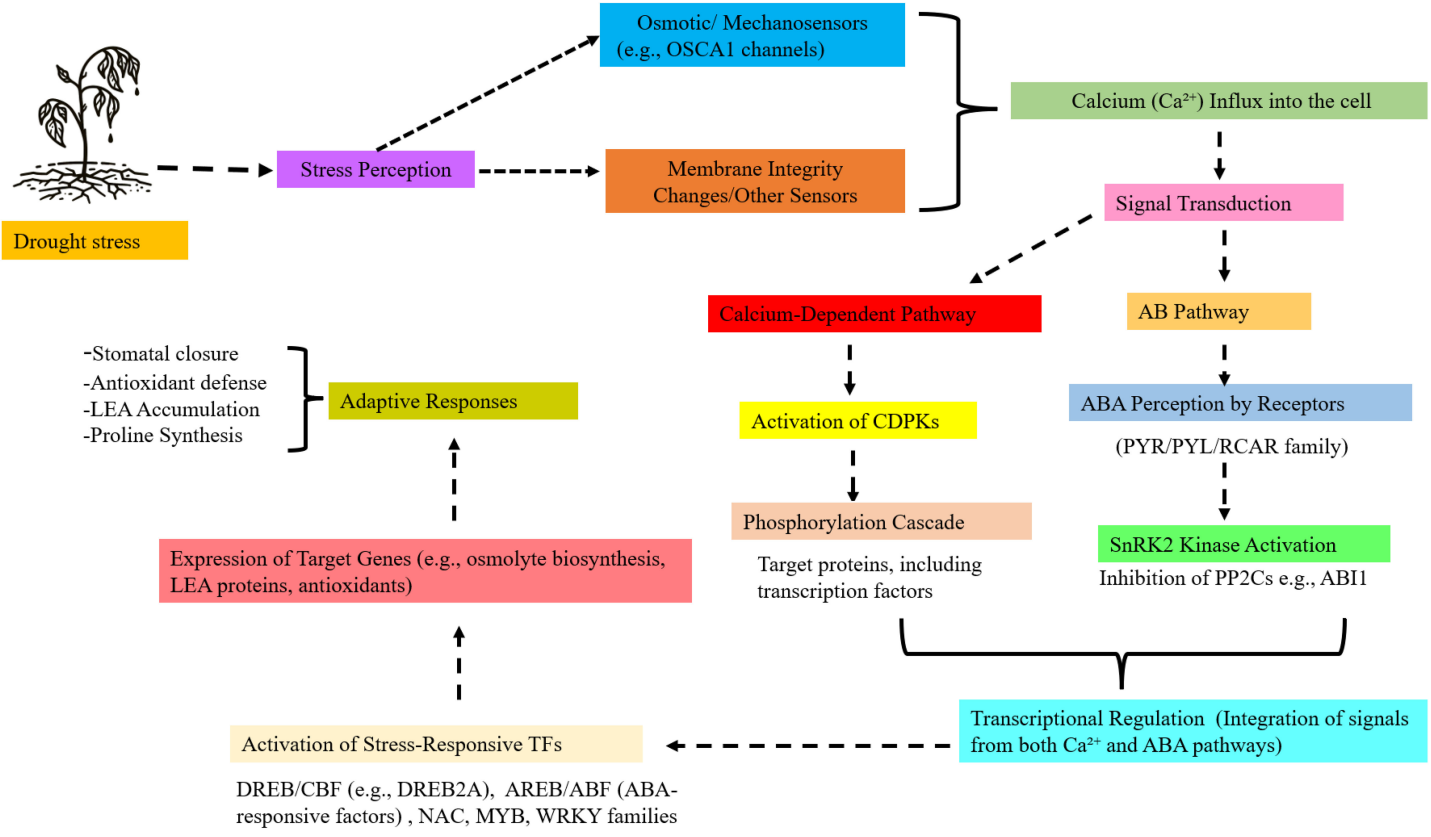
Mechanisms of drought stress response in plants: calcium-dependent protein kinases (CDPKs) pathways and transcriptional regulation. Drought stress is perceived by root systems and triggers osmotic/mechanosensitive signaling such as OSCA1, leading to calcium Ca^2+^ influx into the cell. The rise in cytosolic calcium activates Calcium-dependent protein kinases (CDPKs), which initiate phosphorylation cascades affecting target proteins such as transcription factors. Simultaneously, the abscisic acid (ABA) pathway is engaged through ABA perception by PYR/PYL/RCAR receptor families, resulting in SnRK2 kinase activation and suppression of PP2C phosphatases (e.g., ABI1). Signal transduction from both Ca^2+^ and ABA pathways converge at transcriptional regulation, where the integration of these signals drives the activation of ABA- and Ca^2+^ responsive transcription factors and the expression of stress-adaptive genes. These transcriptional changes promote key adaptive responses, including stomatal closure, antioxidant defense, LEA protein accumulation, and proline synthesis. Dashed arrows indicate cross-talk and regulatory interactions between pathways, highlighting mechanistic convergence in the orchestration of drought tolerance strategies.

Drought stress severely inhibits crop productivity through the impairment of plant water status. Under water limitation, plants trigger complex signal messengers in which CDPKs play an important role in survival. Specific CDPKs isoforms such as *AtCPK10, OsCDPK7, TaCDPK25-U*, and *HvCPK2a* are critically involved in drought tolerance by translating transient increases in cytosolic Ca²^+^ into phosphorylation cascades that regulate stomatal closure, ROS balance, and ABA-mediated signaling. Calcium binding to EF-hand motifs in their calmodulin-like domain induces structural changes that lift autoinhibition, thereby activating kinase function essential for stress adaptation (Mohanta and Sinha, 2016; Singh et al., 2017; Atif et al., 2019; Linghu et al., 2023). Drought induces a rapid mobilization of cytosolic Ca²^+^, which acts as an emergency signal for activating specific CDPKs isoforms. Once activated, these kinases direct a complex adaptive response by phosphorylating downstream substrates, including transcription factors, ion channels, and metabolic enzymes. The integrated signaling and physiological responses mediated by CDPKs under drought stress, as illustrated in **Figure 1**, drought perception activates osmotic and mechanosensors that induce Ca²^+^ influx and ABA signaling, with CDPK−mediated phosphorylation cascades, and SnRK2 activation converging on DREB/CBF, AREB/ABF, NAC, MYB, and WRKY transcription factors to drive the Expression of target genes such as osmolyte, LEA, and antioxidant genes underlying adaptive drought responses. This post-translational regulation modulates gene expression and mediates biological functions crucial for drought tolerance, including stomatal closure, modulation of root growth, and osmotic adjustment. The importance of CDPKs is underscored by their high level of involvement in abscisic acid (ABA) signaling and stress pathways, which centrally integrate a variety of signals into a single defense strategy (Boudsocq and Sheen, 2013; Edel and Kudla, 2016; Su et al., 2024).

### 2.1 Mechanism of drought-induced CDPKs signaling

The molecular mechanism of CDPKs-mediated drought adaptation involves precise signal transduction from calcium perception to physiological output. The primary signal, a drought-induced cytosolic Ca²^+^ burst, activates CDPKs by binding to their calmodulin-like domain, relieving autoinhibition and activating the kinase domain.

A key mechanism is the regulation of stomatal aperture. CDPKs directly phosphorylate components of the stomatal closure machinery. For instance, in *Arabidopsis*, *AtCPK6*, *AtCPK21*, and *AtCPK23* phosphorylate the slow anion channel (SLAC1), facilitating anion efflux and stomatal closure to minimize water loss (Schulz et al., 2013; Maierhofer et al., 2014; Du et al., 2023). This function is conserved across species, as demonstrated by the interaction of *ZmCPK35 and ZmCPK37 w*ith ZmSLAC1 in maize (Li et al., 2022; Du et al., 2023). Similar Ca²^+^–CDPKs–SLAC1 regulatory modules have been observed in tobacco and barley, indicating strong conservation of this mechanism across species (Li and Liu, 2021; Liu et al., 2022). In response to drought stress, *Arabidopsis AtCPK8* phosphorylates a key antioxidant enzyme, CAT3, at Ser-261 to enhance its ability to detoxify ROS and promote the plant’s drought tolerance (Zou et al., 2015). Similarly, rice *OsCPK14* and *OsCPK21* mediated the transcriptional facilitation of the transcription factors (TFs) OsDi19–4 and the regulatory protein OsGF14e, which converge ABA signaling and dry stress response pathways (Wang et al., 2016; Chen et al., 2017).

Moreover, the Calcium-dependent protein kinase *ZmCPK17* phosphorylates and inhibits another maize CDPKs, *ZmCPK2*, under ABA treatment and dehydration conditions (Hu et al., 2024). The inhibition blocks the activating phosphorylation of ZmYAB15, a negative regulator by *ZmCPK2*, derepressing genes responsive to drought (Hu and Xiong, 2014). The interaction of other kinases with CDPKs forms phosphorylation cascades. In *Arabidopsis*, *AtCPK4/11* phosphorylates RBOHD/F, which generates reactive oxygen species (ROS) and defense signaling (Zhao et al., 2021). In tomato (*Solanum lycopersicum*), *SlCDPK2* and *SlCDPK3* regulate drought response by adjusting partially overlapping but different pathways. In water-scarce situations, their synergistic activity helps activate stress-responsive genes, transcription factors, and enzymes, thereby increasing drought tolerance (Hu et al., 2016).

Simultaneously, CDPKs play a crucial role in ABA signaling amplification. They phosphorylate ABA-responsive transcription factors (ABFs), such as ABF1 and ABF4, enhancing the expression of stress-responsive genes involved in osmoprotectant synthesis (e.g., proline) and antioxidant defense (Schulz et al., 2013; Tong et al., 2021; Du et al., 2023). This transcriptional reprogramming is crucial for cellular protection under dehydration stress (Asano et al., 2012).

### 2.2 Transcriptional regulation of CDPKs genes under drought

There is a dynamic regulation of CDPKs gene expression by drought in an isoform-, organ-, and developmental stage-specific manner. The elevation of only a subset of CDPKs suggests that there might be specific functional specializations for distinct drought signaling activities (Yu et al., 2018); not all CDPKs are upregulated, as some are induced quickly, while others are downregulated or remain unchanged.

Noticeable species-specific patterns can be observed in this regulation. For example, in *Arabidopsis*, drought and salinity both trigger the induction of *AtCDPK1* and *AtCDPK2*, but heat- or cold-stress does not (Urao et al., 1994). For example, comparative studies in cereals reveal conservation of expression for some orthologous genes and lineage-specific transcriptional patterns indicative of adaptive evolution to local environments in others (Mittal et al., 2017).

Expression varies greatly by tissue. Some isoforms are predominantly expressed in leaves and guard cells, strongly suggesting a direct relationship with stomatal control. In contrast, others display maximum expression level in roots, which are indispensable for water foraging (Gao et al., 2014; Mittal et al., 2017). The complex transcriptional scenario and potential modulation of subcellular localization (Asano et al., 2012) suggest the importance of studying individual members of CDPKs to understand their specific roles in drought-induced responses.

In addition to these differential expression patterns, drought-induced regulation of CDPKs transcription is governed by complex hormonal and epigenetic control mechanisms. Transcriptional regulation of CDPKs genes during drought stress is governed by both ABA-dependent and ABA-independent signaling pathways. Drought-induced accumulation of abscisic acid (ABA) activates transcription factors such as ABF/AREB, DREB2A, and NAC, which bind to ABRE and DRE motifs in CDPKs promoters, driving isoform-specific gene expression. For example, *GmCDPK5* and *GhCDPK16* are upregulated through ABA pathways, whereas OsCPK9 expression is linked to the enrichment of the ABRE motif involved in stomatal and osmotic regulation (Wei et al., 2014; Veremeichik et al., 2022). In *Zea mays*, *ZmCPK4* and *ZmCPK11* show similar ABA-responsive promoter activity mediated by ABF-binding elements (Borkiewicz et al., 2020). In addition to hormonal control, chromatin remodeling and tissue-specific promoter activation further refine CDPKs transcription. The guard cell-specific expression of *AtCPK1/2* and the root-localized induction of *OsCPK9* exemplify spatially distinct transcriptional responses that support drought adaptation (Hettenhausen et al., 2016; Yan et al., 2024). Together, these multilayered regulatory mechanisms enable the translation of Ca²^+^ signals into precise gene expression patterns, enhancing the plant’s ability to respond to drought in a context-specific manner.

### 2.3 Physiological responses mediated by CDPKs under drought stress

The synergistic signaling pathways of CDPKs underlie important physiological modifications that enhance drought resistance at the whole-plant level. These responses involve the promotion of stomatal closure for reducing water loss, induction of osmotic adjustment through accumulation of solutes such as proline and sugars to support cell turgor maintenance, and the improvement in the protective antioxidant system to deleterious reactive oxygen species (ROS) (Bi et al., 2021; Yan et al., 2022). Additionally, CDPKs also regulate RSA to enhance water absorption from the soil (Uga et al., 2013; Xiong et al., 2021). Transgenic approaches further evidence the functional importance of such adaptations; for example, *OsCPK9* in rice and *GhCPDK16* in cotton, when overexpressed, increase drought tolerance by positively regulating stomatal movement, osmotic adjustment, and antioxidant capability (Wei et al., 2014; Yan et al., 2024). Another tobacco gene, *TaCDPK1-5A*, had higher sensitivity to dehydration and exogenous phytohormone abscisic acid (ABA). The over-expressions (OEs) of the three genes *TaCDPK1-5A*, TaMAPK4-7D, and TaABF1-3A showed improved plant responses to drought by altering physiological parameters (better root morphology and higher rate of stomatal closure) but not osmotic stress, as reported by (Hou et al., 2024).

Recent studies have shown that the CDPKs-related kinases *HvCRK2* and *HvCRK4* function as negative regulators of drought tolerance by interacting with HvCML32, thereby suppressing ABA signaling and promoting ROS accumulation. Their overexpression results in impaired stomatal regulation and diminished antioxidant capacity, underscoring their role in modulating ABA–ROS cross-talk to avoid overactivation of stress responses (Collin et al., 2020; Pan et al., 2022; Cui et al., 2024). These results prove the role of CDPKs as central modulators in drought physiology, and suggest their use as targets for biotechnological crop improvement.

## 3 CDPKs genes and salinity stress

**Figure 2:**
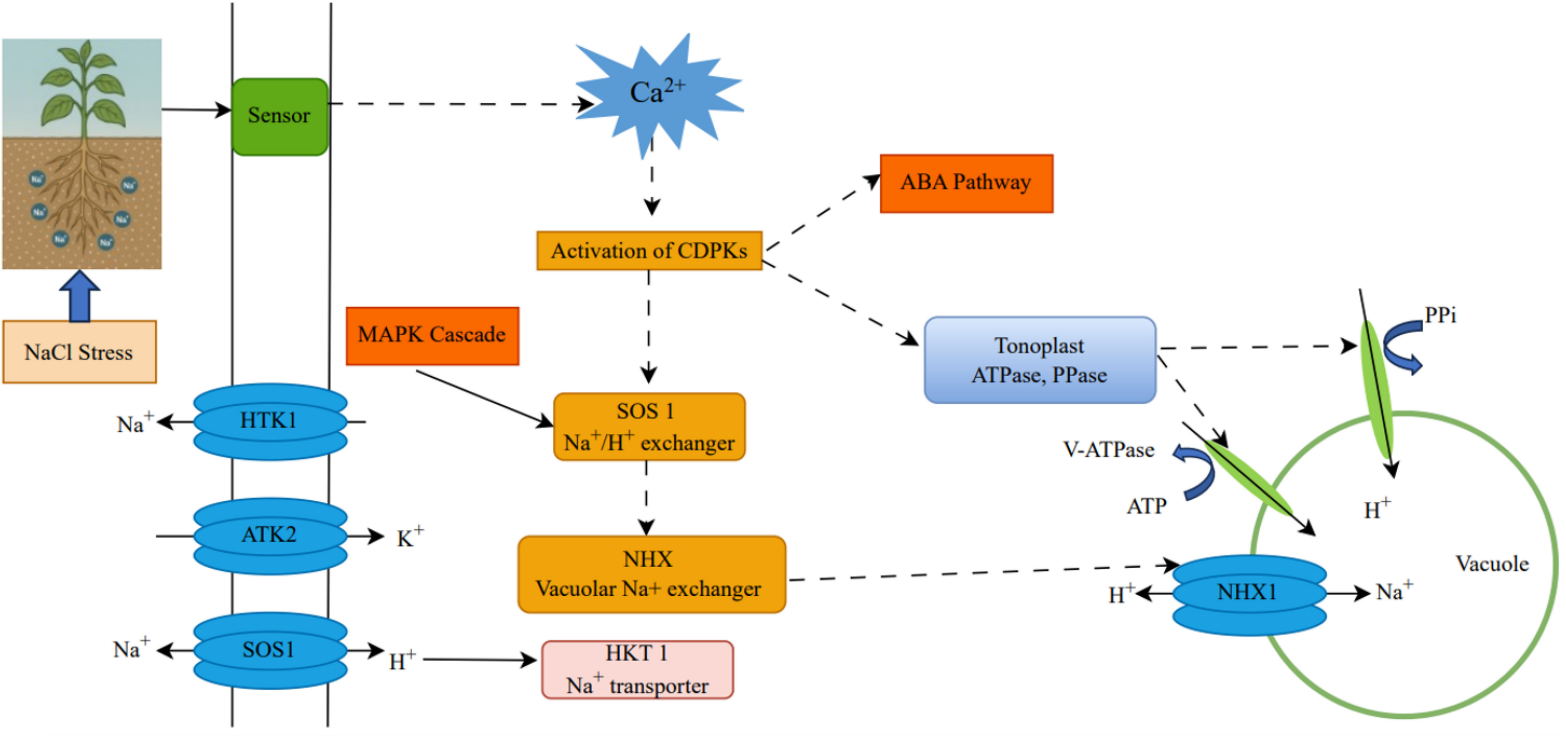
Mechanisms of NaCl stress response in plants. The figure demonstrates the cellular pathways plants use to respond to NaCl stress. High salt concentrations trigger stress sensors to detect NaCl, activating calcium ion (Ca²^+^) signaling to activate Calcium-dependent protein kinases (CDPKs) such as *CmoCDPK20*, *CsCDPK6*, *LpCDPK27*, and *BsCDPK1*. The kinases control essential ion transporters, including SOS1 (Na^+^/H^+^ exchanger), NHX (vacuolar Na^+^ exchanger), HKT1 (Na^+^ transporter), and AKT2 (K^+^ transporter), as well as tonoplast ATPase and pyrophosphatase (PPase) to maintain ion equilibrium. The diagram incorporates the MAPK cascade, which acts in parallel with CDPKs to modulate SOS1 activity and sodium transport. Additionally, the abscisic acid (ABA) signaling pathway interacts with Ca²^+^-responsive processes and CDPKs, influencing tonoplast ATPase and PPase activities to facilitate proton gradient formation and sodium compartmentalization in the vacuole via NHX1. These coordinated signaling modules support efficient Na^+^ exclusion and homeostatic adaptation to salt stress. These coordinated signaling modules support efficient Na^+^ exclusion and homeostatic adaptation to salt stress.

CDPKs are crucial components in the plant signaling system that respond to salinity stress, disrupting cell homeostasis, including osmotic and ionic balance. CDPKs are primarily Ca²^+^ sensors that sense salt-triggered calcium signatures and convert them into specific phosphorylation responses to initiate adaptive reactions (Zhu, 2002; Ludwig et al., 2004). As illustrated in **Figure 2**, salt stress–induced Ca²^+^ signaling activates CDPKs, which, together with the MAPK cascade and ABA pathway, regulate SOS1, NHX, HKT1, and tonoplast ATPase/PPase activities to drive Na^+^ efflux, vacuolar sequestration, and overall ionic homeostasis under NaCl stress.

The transcriptional control of genetic factors under salt stress is also a primary function of CDPKs. They phosphorylate and activate transcription factors, particularly those that regulate gene expression related to ion transport (e.g., SOS pathway components) and the biosynthesis of compatible protective osmolytes, such as proline and glycine betaine (Lehmann et al., 2010; Boudsocq and Sheen, 2013). This signaling is deeply integrated with the abscisic acid (ABA) pathway; ABA-induced CDPKs activation amplifies the stress response, reducing salt damage (Edel and Kudla, 2016). Crucially, CDPKs also directly regulate ion homeostasis by controlling the activity and likely the localization of ion channels and transporters, including the Na^+^/H^+^ antiporters of the SOS system, to reestablish ionic balance (Silva and Gerós, 2009).

The agronomic importance of CDPKs is underscored by their elevated expression and activity in salt-tolerant cultivars (Mittal et al., 2017; Huang et al., 2020). Quantitative assessments in transgenic plants reveal that CDPKs overexpression significantly enhances salt tolerance by promoting ionic homeostasis, ROS scavenging, and osmotic regulation. For instance, overexpression of *TaCDPK27* improved wheat survival by 32% and lowered the Na^+^/K^+^ ratio under 200 mM NaCl stress. Similarly, *CsCDPK6*-expressing lines exhibited a 47% increase in biomass and a 40% reduction in oxidative damage under comparable conditions. Enhanced chlorophyll content and water retention have also been observed in *AtCPK6* and *AkCDPK15* transgenic lines (Atif et al., 2019; Zhu et al., 2021; Yue et al., 2022; Gao et al., 2025). Consequently, transgenic plants overexpressing specific CDPKs genes consistently exhibit enhanced growth and physiological performance in saline environments, confirming their potential as targets for breeding resilient crops.

### 3.1 CDPKs-mediated mechanisms of salinity tolerance

Salinity inflicts damage through a tripartite mechanism: osmotic stress, which impedes water uptake; ionic Stress, caused by Na^+^ and Cl^-^ accumulation that disrupts enzyme function and membrane integrity; and secondary oxidative Stress resulting from the generation of reactive oxygen species (ROS) (Kaushal, 2020; Van Zelm et al., 2020). CDPKs orchestrate a concerted defense against these challenges to maintain cellular homeostasis. In rice, *OsCPK5* and *OsCPK13* exemplify such specialization. *OsCPK5* functions as a positive regulator of salt stress tolerance by phosphorylating and activating mitogen-activated protein kinases (MAPKs) such as OsMPK3 and OsMPK6, which have a role in potassium signaling under saline environments (Su et al., 2024).

Firstly, Ion Homeostasis: CDPKs are pivotal in regulating ionic balance, primarily through the SOS pathway. They are implicated in signaling that activates Na^+^/H^+^ antiporters for Na^+^ extrusion and vacuolar sequestration, while also potentially regulating K^+^ channels to maintain a favorable K^+^/Na^+^ ratio, a critical determinant of salt tolerance (Boudsocq and Sheen, 2013; Edel and Kudla, 2016). In *Arabidopsis*, isoforms like *AtCPK12* and *AtCPK23* are directly involved in Na^+^ regulation (Zhang et al., 2018).

Moreover, Osmotic Adjustment: CDPKs drive the accumulation of compatible solutes to counteract osmotic stress. They promote the biosynthesis of osmolytes, such as proline (e.g., via *AtCPK1*) and glycine betaine, which help maintain cell turgor and protect cellular structures without interfering with metabolic processes (Urao et al., 1994; Lehmann et al., 2010). Furthermore, Oxidative Stress Mitigation: CDPKs activate the antioxidant defense system to mitigate ROS-induced damage. Functional studies reveal that *ZmCPK11* regulates ROS detoxification via phosphorylation cascades that enhance SOD, APX, and CAT activities under stress. Acting upstream of ZmMPK5, it mediates ABA-induced antioxidant responses and limits damage caused by lipid peroxidation. Overexpression of *ZmCPK11* increased SOD activity by 38% and reduced H_2_O_2_ accumulation by 40% in transgenic lines, confirming its pivotal role in Ca²^+^–ABA–ROS signaling. Similar redox-modulatory functions are shared by related isoforms *ZmCPK17* and *ZmCPK7* (Ding et al., 2013; Borkiewicz et al., 2020; Hu et al., 2024).

Functional studies in diverse species validate the efficacy of this CDPKs-mediated network. Overexpression of *BsCDPK1*, *CmoCDPK20*, *CsCDPK6*, and *LpCDPK27* (Zhu et al., 2021; Su et al., 2024; Chen et al., 2025; Wang et al., 2025) consistently improves salt tolerance phenotypes, including better germination, root growth, ion homeostasis (elevated K^+^/Na^+^ ratio), reduced oxidative markers (MDA, H_2_O_2_), and enhanced expression of stress-responsive genes. In summary, CDPKs signaling consolidates the regulation of ion transport, osmolyte production, and ROS detoxification into a unified adaptive response. The central regulatory function of CDPKs positions them as optimal targets for biotechnological approaches focused on cultivating crops for sustainable agriculture in saline conditions.

## 4 CDPKs genes and temperature stress

CDPKs play pivotal roles in the complex signal transduction pathways to heat and cold stress. As the primary Ca²^+^ sensors, CDPKs transform temperature-generated alterations in cytosolic Ca²^+^ levels into distinct phosphorylation statuses, thereby integrating multiple protective physiological processes. As the planet warms and temperatures shift more frequently, the importance of these factors is growing. High and low temperatures disrupt native cell processes by denaturing proteins, breaking down membranes, and increasing reactive oxygen species (ROS), among other effects. CDPKs mediate the restoration of damage by modulating key processes, including the upregulation of heat shock proteins (HSPs), activation of antioxidant systems, and maintenance of membrane stability and osmotic balance. This increases the probability of survival for plants (Petrov et al., 2015; Zhao et al., 2017).

### 4.1 Response of CDPKs genes to heat stress

**Figure 3:**
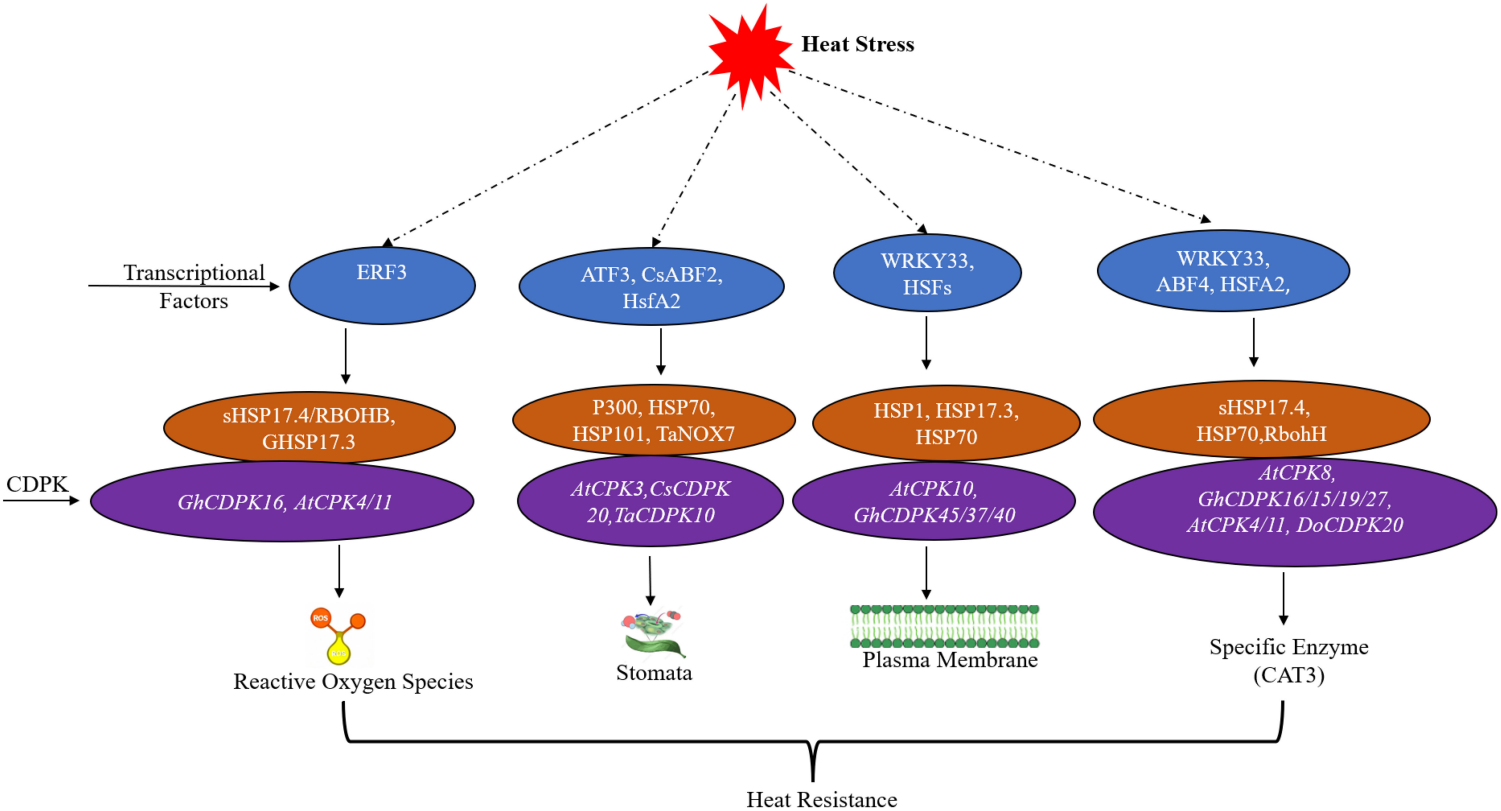
Mechanisms of heat stress response and resistance in plants. The figure illustrates plant heat stress responses through molecular mechanisms, demonstrating how transcription factors and Calcium-dependent protein kinases (CDPKs) function in signaling pathways to regulate heat tolerance. The transcription factors ERF3, ATF3, CsABF2, HsfA2, WRKY33, HSFs, and ABF4 initiate stress-responsive gene expression when plants encounter elevated temperatures. The CDPKs *GhCDPK16, AtCDPK4/11, AtCPK3, CsCDPK20, TaCDPK10, AtCPK10, GhCDPK45/37/40, AtCPK8*, and *GhCDPK16/15/19/27* function through phosphorylation to control stomatal function as well as plasma membrane stability and enzymatic activity. For instance, *AtCPK3* activates S-type anion channels, such as SLAC1, in guard cells when it receives signals from calcium. This makes it easier for ions to leave the cell and for the stomata to shut. During ethylene signaling, *AtCPK3* also makes more reactive oxygen species (ROS) through NADPH oxidases (AtRBOHD/F). It also helps close stomata when pathogens are present by chance (Mori et al., 2006; Boudsocq and Sheen, 2013; Zhang et al., 2022). *TaCDPK10* is a kinase in guard cells that senses Ca²^+^ and controls the activity of inward K^+^ channels. It also helps close stomata to lower transpiration. Cellular protection against thermal damage depends on reactive oxygen species (ROS), small heat shock proteins (sHSPs), and antioxidant enzymes, including catalase (CAT3). Together, these mechanisms help plants survive heat stress conditions, and scientists can use them to develop molecular approaches to enhance crop thermotolerance.

Activation of the CDPKs genes, as illustrated in **Figure 3**, heat stress activates specific transcription factors and heat−shock protein modules that are regulated by CDPKs, thereby modulating ROS production, stomatal function, plasma−membrane stability, and antioxidant enzymes to collectively enhance heat resistance, is an important early step in heat shock signal transduction caused by the disruption of intracellular calcium concentrations. Increased temperature would induce an influx of Ca²^+^ ions that activate specific CDPKs isoforms that, in turn, transduce this signal through restricted phosphorylation cascades to induce the core cascade of stress-responsive pathways (Klimecka and Muszyńska, 2007; Yang et al., 2021). One primary function of the CDPKs during heat stress is their direct modulation, achieved by binding to and modifying factors involved in the heat-shock response. The small heat shock protein sHSP17 is a substrate of *ZmCPK7—4* at Ser-44, affecting its chaperone activity and general thermotolerance. The same kinase phosphorylates the respiratory burst oxidase homologue RBOHB at Ser-99, implying that 118 is involved in the modulation of ROS signaling under Stress (Zhao et al., 2021). Physical interaction of *OsCPK12* with OsHSP18. 0 results in phosphorylation of Thr-32 and increases the protein stability against thermal inactivation (Chen et al., 2011; Kuang et al., 2017).

CDPKs regulate the heat shock transcriptional program. *AtCPK3* and -13 have been shown to phosphorylate the HSFB2a transcription factor, providing an indirect level of control over SP synthesis by CDPKs (Du et al., 2023). This regulation of CDPKs, as part of signaling cascades, contributes to the overall complexity of hormone and ROS signal transduction networks. This coordinated response is reinforced by the interplay of different signaling pathways, such as regulation of gene expression, activation of antioxidant enzymes, and stomatal conductance modulation, contributing to a solid global achievement of thermotolerance (Saidi et al., 2009; Zou et al., 2010; Schulz et al., 2013; Atif et al., 2019). This regulatory role is vital, as studies have shown that the elevated expression of specific CDPKs isoforms directly correlates with enhanced heat stress tolerance.

### 4.2 Cold stress and CDPKs gene regulation

**Figure 4:**
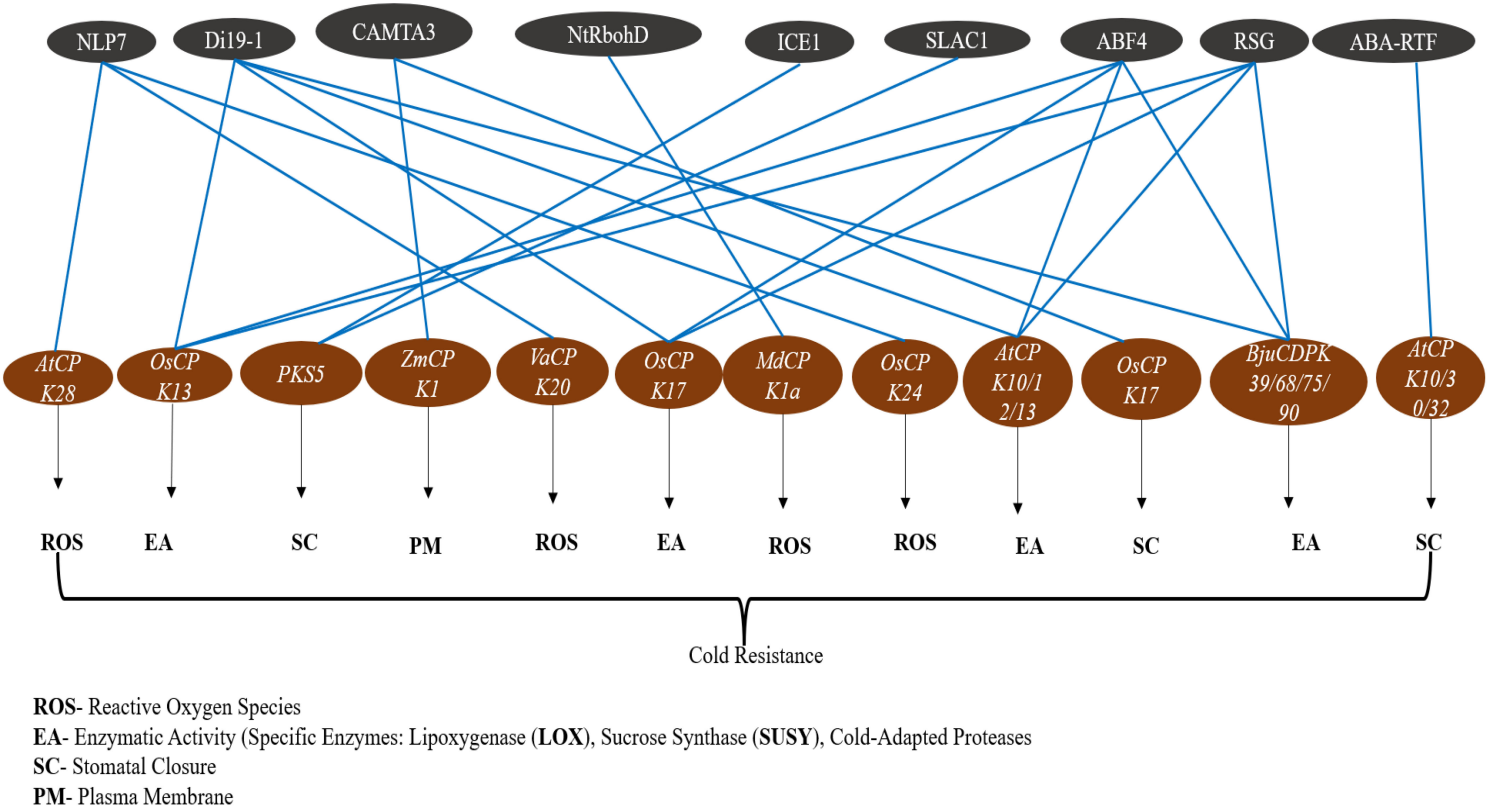
Interactions between Calcium-dependent protein kinases (CDPKs) and transcription factors in cold stress pathways. This figure illustrates the complex regulatory network between Calcium-dependent protein kinases (CDPKs) and transcription factors involved in cold stress adaptation in plants. Functional categories include reactive oxygen species (ROS), environmental adaptation (EA), stomatal closure (SC), and plant metabolism (PM), each of which contributes to plant resilience under cold conditions. Blue connecting lines represent documented interactions between transcription factors and CDPKs, highlighting their roles in cold stress signaling and adaptation.

CDPKs are crucial regulatory factors for plant cold tolerance, acting as key calcium sensors and signal transducers in response to low temperatures. **Figure 4** depicts the interaction network between CDPKs and transcription factors involved in cold stress adaptation via the plasma membrane, enzymatic activities (lipoxygenase), and reactive oxygen species. A rapid array of physiological responses is induced by cold, most notably a prominent transient increase in intracellular calcium contents, an initial signal for CDPKs activation (Iqbal et al., 2022). Several CDPKs isoforms, such as *AtCPK32*, are induced and activated at higher levels during cold stress, suggesting their specific functions in nucleic acid regulation for conferring increased stress tolerance. Conversely, *OsCPK13* responds to cold and gibberellin (GA) signals, participating in stress signaling by phosphorylating transcription factors, including OsERF104. Therefore, augmenting the expression of genes related to cold tolerance (Abbasi et al., 2004).

These kinases exert significant control over the cold acclimation adaptive process, influencing gene transcription, metabolism, and the antioxidative response with considerable strength, thereby providing a high level of protection against oxidative stress-induced injury by low temperatures. It is well-documented that CDPKs activity is closely associated with the CBF/DREB transcriptional cascade, which controls the expression of several cold-responsive genes and thus plays an essential role in cold adaptation (Yuan et al., 2018; Tong et al., 2021). By contrast, *ZmCPK1-a*, a calcium-independent maize CDPKs, downregulates cold tolerance by decreasing the expression of cold-induced ZmERF3 and therefore can act as a negative modulator in the fine-tuning of cold stress signaling (Weckwerth et al., 2015). CDPKs mediate a complex cross-talk with reactive oxygen species (ROS).

However, cold shock often accumulates pro-inflammatory and potentially harmful ROS; these molecules also act as secondary messengers. CDPKs connect the ROS signal to the stress response network, enabling plants to cope with low temperatures by regulating osmotic balance, detoxifying ROS, and repairing cellular damage (Dreyer and Dietz, 2018; Dong et al., 2020; Shi and Zhu, 2024). They maintain membrane fluidity and ion balance to ensure cell membrane stability under cold stress by maintaining physiological homeostasis. The activity of specific isoforms relies on their subcellular localization, which can alter in response to low temperatures, as has been shown for *AtCPK32*, emphasizing the importance of spatiotemporal specificity in their function as regulators of gene expression (Zou et al., 2010; Atif et al., 2019). CDPKs function as key factors to enable a coherent and effective transcriptional and physiological response to cold, consolidating their role as master regulators of cold acclimation and tolerance in plants through physical or genetic interactions with a variety of downstream targets, as well as hormonal signaling cascades such as abscisic acid (ABA).

## 5 Cross-talk: CDPKs at the junction of multiple stress signaling pathways

**Figure 5:**
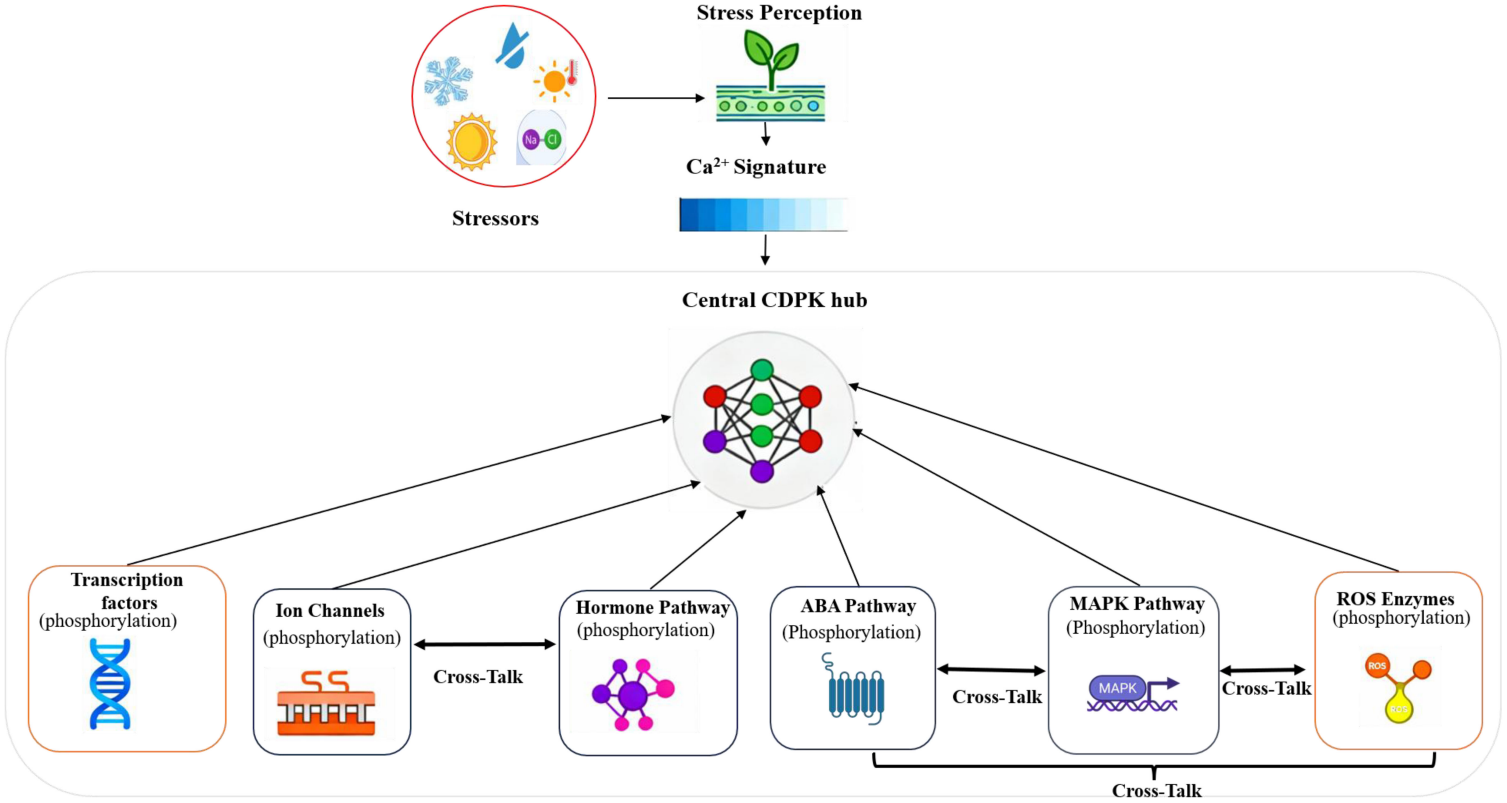
Schematic representation of Calcium-dependent protein kinases (CDPKs) as a central hub of plant abiotic stress signaling. Diverse abiotic stressors, including cold, drought, heat, oxidative stress, and salinity, are perceived by plant cells, eliciting distinct Ca²^+^ signaling signatures. These Ca²^+^ signals activate the central CDPKs hub, which transduces stress signals through phosphorylation of multiple downstream targets. Key CDPKs-regulated components include transcription factors, ion channels, hormone-related pathways (including ABA signaling), MAPK cascades, and enzymes associated with reactive oxygen species (ROS) homeostasis. Extensive cross-talk between these modules enables dynamic modulation of gene expression, membrane transport, hormonal balance, and oxidative stress responses, orchestrating rapid and coordinated cellular adaptation to environmental challenges.

Plants frequently experience simultaneous abiotic stressors, including dryness and salinity or heat and drought. They have developed intricate signaling networks to address these issues, in which CDPKs are key integrators. As illustrated in **Figure 5**, the central CDPKs hub integrates diverse stress-induced Ca²^+^ signatures and mediates downstream phosphorylation of transcription factors, ion channels, hormone pathways, and ROS enzymes, coordinating cross-talk among these signaling pathways to regulate plant stress responses. CDPKs serve as molecular interpreters that translate stress-specific calcium signals into precise physiological responses by phosphorylating several downstream substrates, facilitating coordinated adaptation to numerous stresses (Ludwig et al., 2004).

CDPKs function as pivotal integrators of plant responses to diverse abiotic stresses by mediating Calcium-dependent protein kinases regulation of hormonal signaling, redox balance, and ion homeostasis. In rice, isoforms such as *OsCPK4*, *OsCPK7*, *OsCPK12*, *OsCPK13*, *OsCPK17*, *OsCPK21*, and *OsCPK26* operate synergistically under combined drought, salinity, and cold stress conditions. These kinases promote cross-tolerance by activating antioxidant enzymes, preserving plasma membrane stability, and modulating ABA-responsive transcriptional networks (Asano et al., 2011; Mohanta and Sinha, 2016; Naeem et al., 2019). Similarly, in *Arabidopsis*, *AtCPK10, AtCPK11, AtCPK23*, and *AtCPK32* contribute to drought, salt, and cold Stress tolerance by regulating stomatal dynamics and inducing oxidative stress–responsive genes via ABA and MAPK signaling pathways (Asano et al., 2012; Gao et al., 2014; Naeem et al., 2019; Akram et al., 2024). This signaling complexity is further amplified by cross-talk with MAPK cascades; for instance, *AtCPK5* activates the NADPH oxidase RBOHD to generate ROS, which in turn amplifies the stress signal through MAPK activation (Dubiella et al., 2013).

In *Zea mays*, *ZmCPK4* and *ZmCPK17* orchestrate drought–heat stress responses by enhancing the expression of ROS-scavenging enzymes and heat shock transcription factors, thereby sustaining photosynthetic performance and cellular stability under elevated temperatures (Du et al., 2023; Hu et al., 2024). In *Gossypium hirsutum*, *GhCPK33* exemplifies a multifunctional CDPKs, conferring resistance to salt, heat, and oxidative stress through upregulation of antioxidant defenses and osmoprotectant-related genes (Akram et al., 2024; Patil et al., 2024). Cotton utilizes *GhCPK1*, *GhCPK6*, and *GhCPK23* to modulate ion transporters under saline-drought conditions (Li et al., 2015; Mahmood et al., 2019; Wang et al., 2024). Mulberry enhances salt-drought tolerance through the regulation of stress-responsive genes by *MaCDPK1* and *MaCDPK10* (Cao et al., 2020). Collectively, these studies highlight the remarkable functional versatility of CDPKs in mediating cross-tolerance. Distinct isoforms function as calcium-responsive signaling nodes, converting transient calcium signatures into targeted phosphorylation events that integrate ABA signaling, ROS detoxification, and ionic regulation under complex environmental stress combinations (Dutta et al., 2024; Patil et al., 2024).

The integration of CDPKs signaling with ABA pathways is a cornerstone of cross-tolerance. In rice, this is exemplified by OsMADS23-mediated upregulation of both ABA biosynthesis genes (OsNCED2/3/4) and the proline synthesis gene OsP5CR (Li et al., 2023). These findings collectively position CDPKs as central hubs in Stress signaling networks, making them promising targets for engineering multi-stress-resistant crops. Multi-omics investigations provide compelling evidence for the integration of CDPKs-mediated ion and redox signaling under combined abiotic stress conditions. In *Zea mays*, transcriptomic analyses revealed the coordinated upregulation of *ZmCPK11*, *ZmCPK17*, and *ZmCPK37* alongside ROS-scavenging genes during concurrent drought and heat stress (Du et al., 2023). Comparable transcriptomic and proteomic studies in *Cicer arietinum* and *Fragaria × ananassa* demonstrated co-expression of CDPKs with antioxidant enzymes under salt–heat and salt–cold stress combinations, respectively (Crizel et al., 2020; Deepika et al., 2022). A recent comparative genomic analysis in *Populus* identified conserved CDPKs gene clusters associated with redox and ion homeostasis under stress conditions (Mu et al., 2024).

Additionally, proteomic interaction network mapping has highlighted central roles for CDPKs in orchestrating cross-talk among Ca²^+^, ROS, and ABA signaling pathways during complex stress responses (Zubair et al., 2025). Current research focuses on manipulating key CDPKs nodes, such as *OsCPK12* for ROS management and *GhCPK1* for ion homeostasis, through genetic engineering to develop broad-spectrum climate resilience. This approach leverages the conserved yet adaptable nature of the CDPKs signaling family.

Multiple CDPKs–substrate phosphorylation events have been experimentally validated, offering mechanistic insights into how CDPKs translate Ca²^+^ signals into stress-responsive outcomes across plant species. **Table 3** provides a consolidated summary of these experimentally characterized CDPKs targets, detailing their phosphorylation sites, stress contexts, and physiological consequences. These examples highlight the conserved yet functionally specialized roles of CDPKs isoforms in fine-tuning the adaptation to drought, salinity, heat, and oxidative stress.

**Table 3:** Experimentally validated CDPKs substrates, phosphorylation sites, and physiological outcomes under abiotic stress.

CDPKs isoform	Substrate protein	Phosphorylation site(s)	Stress context	Validated function/physiological outcome	Reference
*AtCPK21, AtCPK23*	SLAC1 (slow anion channel 1)	Ser^59^, Ser^120^	Drought	Activation of anion efflux, promoting stomatal closure	(Brandt et al., 2012; Maierhofer et al., 2014)
*AtCPK5, AtCPK6, AtCPK11*	RBOHD (NADPH oxidase)	Ser^148^, Ser^343^, Ser^347^	Pathogen (PAMP) & abiotic cues	Phosphorylation activates RBOHD → ROS burst and systemic defense/signaling	(Dubiella et al., 2013; Kadota et al., 2014)
*OsCPK12*	*OsSLAC1*	Ser¹^6^³, **S**11 on OsCATA/OsCATC	Drought, salt, oxidative stress	Na^+^ efflux, osmotic balance.	(Asano et al., 2012; Wang et al., 2024)
*OsCPK21*	*OsGF14e* (14-3–3 protein)	Tyr138	Salt	Modulates ABA− and salt−responsive gene expression through Ca²^+^−dependent phosphorylation of 14−3−3 proteins.	(Chen et al., 2017)
*OsCPK9*	Plasma membrane H^+^-ATPase (AHA)	Ser^955^	Drought	Regulation of ion transport and stomatal conductance.	(Wei et al., 2014)
*ZmCPK17*	*ZmCPK2* (CPK interactor) and *ZmYAB15* (TF)	*ZmCPK2* T60 (CPK17 phosphorylates *ZmCPK2* at T60)	Drought	*ZmCPK17* phosphorylates or inhibits *ZmCPK2* and regulates downstream TF (*ZmYAB15*) → promotes drought adaptation.	(Hu et al., 2024)
*TaCDPK2,/TaCDPK21*	Plasma membrane channels, MAPKs	not mapped	Cold, Drought	Ion flux control, ROS signaling.	(Arefian et al., 2021)
*TaCDPK5*	TabZIP60 (bZIP TF)	not mapped	Abiotic stress, ER-stress-related pathways.	*TaCDPK5* (and *TaCDPK9-1*) phosphorylate TabZIP60 → regulation of stress-responsive transcription.	(Zhang et al., 2022)
*GhCDPK16*	SOD, CAT (ROS-scavenging enzymes)	not mapped	Drought, Heat	Enhanced ROS detoxification and antioxidant activity.	(Yan et al., 2024)
*LeCDPK2*	ACS (1-Aminocyclopropane-1-carboxylic acid synthase)	Ser^107^	Heat, Drought	Modulation of ethylene biosynthesis.	(Dekomah et al., 2022)
*StCDPK7*	Autophosphorylation & downstream TFs	not mapped	Drought	Stress-induced phosphorylation, ROS homeostasis.	

This table compiles experimentally confirmed CDPKs–substrate phosphorylation events from multiple plant species, summarizing their validated target proteins, phosphorylation sites (where identified), stress contexts, and resulting physiological or transcriptional outcomes.

## 6 Research controversies and unexplored aspects

Whereas many family members are positive regulators in plant abiotic stress response, there is now evidence that some isoforms can also act as negative regulators, underscoring the complex and context-dependent nature of CDPKs activity. The functional duality of CDPKs in plant stress signaling is highly dependent on cellular context, dictated by isoform-specific expression patterns, post-translational modifications (PTMs), and dynamic protein–protein interactions. Isoforms such as *AtCPK1*, *AtCPK23*, and *OsCPK12* exhibit contrasting regulatory roles in ROS and ion signaling pathways, which are influenced by their subcellular localization and phosphorylation state. For example, autophosphorylation of *AtCPK1* enhances its activity toward the NADPH oxidase RBOHD, facilitating ROS production during the early phases of stress. In contrast, S-nitrosylation of CDPKs under sustained oxidative conditions inhibits kinase activity, thereby preventing excessive ROS accumulation.

Additionally, ubiquitin-dependent degradation of *OsCPK17* has been shown to modulate the duration of its signaling activity under combined salt and heat stress. These context-specific regulatory mechanisms, together with interactions involving 14-3–3 proteins, MAPKs, and calmodulin-like proteins, allow individual CDPKs to function as both positive and negative regulators of stress responses. This enables precise interpretation of Ca²^+^ signals and ensures appropriate physiological outcomes under fluctuating environmental conditions (Paul et al., 2005; Boudsocq and Sheen, 2013; Zubair et al., 2025). For example, *AtCPK23* shifts from acting as a positive to a negative regulator of guard-cell signaling as the strength and duration of calcium oscillations rise. It activates SLAC1 to promote stomatal closure under moderate ABA levels, but later suppresses inward K^+^ channels (*GORK*) during extended stress to prevent excessive ion loss (Zou et al., 2010). The roles of individual CDPKs can differ significantly even within the same species. *AtCPK10* is a positive regulator of *Arabidopsis thaliana* by mediating ABA- and Ca²^+^-induced stomatal closure (Zou et al., 2010).

In contrast, *AtCPK23* is a flexible gene that can function as either a positive or negative regulator of stomatal closure (Ma and Wu, 2007; Atif et al., 2019). Barley *HvCRK2* and *HvCRK4* suppress drought responses by abrogating ABA-response pathways and activating ROS accumulation. This renders them repressors of dehydration tolerance (Cui et al., 2024). This functional divergence is particularly pronounced in maize, with members of the CDPKs family serving distinct functions. *ZmCPK4*, *ZmCPK11*, *ZmCPK35*, and *ZMCPK37* function as positive regulators in response to drought and salinity challenges, which are exerted through ABA-mediated stomatal closure, maintenance of Na^+^/K^+^ homeostasis, and protection of the photosynthetic process (Jiang et al., 2013; Borkiewicz et al., 2020; Li et al., 2022). Likewise, in maize, *ZmCRK1* demonstrates a stress-intensity-dependent regulatory shift, where phosphorylation of the plasma membrane H^+^-ATPase ZmMHA2 suppresses proton-driven solute uptake during severe drought, yet enhances ion homeostasis and antioxidant defense under milder stress conditions (Liu et al., 2024).

These dual or opposing regulatory roles are supported by genetic and biochemical approaches, including loss-of-function and overexpression mutants, kinase activity assays, and phosphoproteomic analyses (Zou et al., 2010; Hu et al., 2024). Mechanistically, such context dependency arises from isoform-specific Ca²^+^ sensitivities, subcellular localization patterns, and post-translational modifications (e.g., autophosphorylation and S-nitrosylation), which influence CDPKs–substrate interactions under different environmental or hormonal contexts (Schulz et al., 2013). Consequently, CDPKs act as conditional signal modulators rather than strictly positive or negative regulators, adjusting their activity according to stress intensity, Ca²^+^ amplitude, and tissue type.

The functional ambiguity of CDPKs extends to stress-specific responses, as exemplified by rice *OsCPK12*, which enhances salt stress tolerance but may compromise resistance to blast disease, suggesting stress-specific functional trade-offs (Asano et al., 2012). Similarly, in cowpea, *VuCPK11* and *VuCPK19* regulate drought and salinity responses through transcriptional coordination with VuDREB2A, implicating these kinases as candidate targets for genetic engineering of multi-stress tolerance (Punniyamoorthy and Jegadeesan, 2025). This stress-specific specialization provides a promising framework for crop genetic engineering, where selective overexpression of CDPKs isoforms such as *OsCPK4*, *AtCPK10*, or *ZmCPK11* has been shown to improve drought, salinity, and heat tolerance without yield penalties, demonstrating the potential to fine-tune stress adaptability through isoform-guided gene editing (Priyadarshan, 2019; Khan, 2025).

Understanding stress-specific CDPKs specialization not only refines our mechanistic insight into Ca²^+^ signal decoding but also opens avenues for precision engineering of crop resilience, where isoform selection and promoter customization can optimize multi-stress tolerance. This complexity necessitates carefully characterizing individual CDPKs isoforms across different stress conditions to fully understand their roles in plant stress adaptation fully. Future research should integrate isoform-specific, multi-omics, and combinatorial Stress analyses to unravel CDPKs functional diversity. Differences in phosphorylation motifs, localization, and interactions drive isoform specialization, which can be resolved through single-cell transcriptomics and phosphoproteomics (Wu et al., 2025). These integrated approaches will clarify CDPKs signaling hierarchies and enable systems-level genetic engineering for multi-stress tolerance in crops.

### 6.1 Functional redundancy within the CDPKs family

Functional redundancy among CDPKs isoforms constitutes a key evolutionary mechanism that ensures both signaling resilience and adaptive versatility under stress conditions. Many CDPKs exhibit high sequence similarity, shared Ca²^+^-binding properties, and overlapping substrate specificities, allowing for compensatory activation when specific isoforms are disrupted. For example, *AtCPK4* and *AtCPK11* redundantly phosphorylate ABF transcription factors within the ABA signaling pathway, safeguarding stress-induced gene expression in the event of single isoform loss (Zhu et al., 2007). Likewise*, AtCPK5* and *AtCPK6* cooperatively activate RBOHD to sustain ROS production during pathogen attack and salt stress (Schulz et al., 2013). In rice, *OsCPK4* and *OsCPK21* exhibit partial functional overlap in modulating ion balance and ROS detoxification under drought and salinity stress (Atif et al., 2019; Wang et al., 2025).

Biologically, this redundancy confers robustness to signaling networks, ensuring continuity and buffering against systemic failure during overlapping or fluctuating environmental stresses. However, from a functional genomics perspective, such redundancy complicates the characterization of genes. Single-gene knockout mutants often show no discernible phenotype due to compensatory expression of paralogous isoforms. At the same time, overexpression studies may yield misleading results due to unintended cross-activation of related CDPKs. To overcome these challenges, recent approaches include CRISPR/Cas9-mediated multiplex knockouts (e.g., *AtCPK4/11* double mutants), the use of isoform-specific transcriptional reporters, and phosphoproteomic analyses to delineate primary signaling roles from compensatory effects (Wang et al., 2020).

Collectively, these findings suggest that CDPKs redundancy extends beyond genetic similarity, representing a structured regulatory feature that enables the precise decoding of calcium signals and the flexible coordination of stress responses across diverse environmental contexts.

## 7 Conclusion

This review has synthesized the compelling evidence that CDPKs are master regulators of plant abiotic stress signaling. As primary decoders of stress-induced calcium signatures, CDPKs orchestrate a sophisticated phosphorylation network that integrates environmental cues into precise physiological adaptations. It detailed their pivotal role in mitigating the effects of drought, salinity, and temperature extremes by regulating key processes such as stomatal aperture, ion homeostasis (e.g., through the SOS pathway), osmotic adjustment via osmolyte accumulation, and antioxidant defense against reactive oxygen species (ROS). The extensive cross-talk between CDPKs and phytohormone pathways, particularly ABA, underscores their role as central hubs that amplify and specify stress signals.

A critical emerging theme is the remarkable functional diversity and context-dependency within CDPKs families. While many isoforms are potent positive regulators of stress tolerance, others, such as *ZmCRK1* in maize and *HvCRK2/4* in barley, function as negative regulators. These duality, functional redundancy, and specificity highlight the complexity and fine-tuning of CDPKs signaling networks. Their ability to confer cross-tolerance to combined stresses further emphasizes their evolutionary significance as integrators of multiple environmental signals. The conservation of CDPKs functions across diverse plant species, from *Arabidopsis* to major crops like rice, maize, and cotton, solidifies their fundamental role in plant adaptation and their immense potential as targets for biotechnological intervention.

## 8 Future perspectives

Despite significant progress, several research avenues are crucial to fully harnessing the potential of CDPKs for crop improvement. Future work must focus on deciphering isoform-specific functions by employing advanced CRISPR/Cas-based gene editing and single-cell transcriptomics to delineate the unique, redundant, and antagonistic roles of individual CDPKs isoforms within specific cell types and developmental stages. Concurrently, a significant effort is needed to elucidate the complete phosphorylation network through phosphoproteomics and biochemical assays to map all downstream substrates, including transcription factors and metabolic enzymes. Furthermore, understanding the context-dependency of CDPKs actions and their cross-talk with other kinase families and hormone pathways beyond ABA is essential for accurate network modeling. Translational research should then leverage this knowledge by exploring sophisticated strategies such as stress-inducible and tissue-specific promoters, stacking favorable alleles while silencing negative regulators, and utilizing synthetic biology to design enhanced CDPKs variants. Finally, exploring natural variation in crop germplasms through GWAS will identify superior alleles for breeding. By integrating these fundamental and applied strategies, we can precisely engineer CDPKs networks to develop climate-resilient crops without growth penalties, thereby securing future food production.

Despite substantial progress having been made in understanding CDPKs-mediated stress responses and applying genome editing for precise functional studies, several obstacles remain. A key drawback of CRISPR/Cas systems is the risk of off-target mutations, which may cause unintended genetic changes or subtle transcriptomic shifts that affect long-term development. Moreover, complex and pleiotropic stress responses, especially under combined stress conditions, can obscure genotype-phenotype relationships, as overlapping pathways or isoform redundancy often conceal the specific effects of individual genes. To overcome these challenges, future research should employ particular guide RNA design, comprehensive off-target detection strategies, and integrative phenomics analyses to enhance the precision and reliability of CDPKs-based crop engineering.
